# Prevalence of *Tungiasis* and its risk factors of among children of Mettu woreda, southwest Ethiopia, 2020

**DOI:** 10.1371/journal.pone.0262168

**Published:** 2022-01-05

**Authors:** Sime Daba Jorga, Yohannes Lulu Dessie, Mohammed Reshad Kedir, Dereje Oljira Donacho

**Affiliations:** 1 Department of Public Health, College of Health Science, Mettu University, Metu, Ethiopia; 2 Department of Nursing, College of Health Science, Mettu University, Metu, Ethiopia; 3 Department of Health Informatics, College of Health Science, Mettu University or Department of Environmental Health Science and Technology, Jimma University, Jimma, Ethiopia; San Giuseppe Hospital, ITALY

## Abstract

Tungiasis is caused by the flea tunga penetrans and results in painful skin lesions, skin infections, and permanent disability. However, limited information is available that shows the magnitude of the problem and its risk factors that help for intervention in Ethiopia. The goal of this study was to determine the prevalence and risk factors of tungiasis in children aged 5 to 14 in Mettu woreda in 2020. A community based cross sectional study was conducted among randomly selected kebeles of Mettu woreda, in Southwest Ethiopia. To select study participants, multistage sampling was used. The data were collected through physical examination of the children, interview of parents/guardians of the children, and observation of the home environment using checklists and questionnaires. The descriptive analysis was done for socio-demographic characteristics, prevalence of tungiasis, and housing conditions. A logistic regression analysis was performed, and variables in multivariable regression reported odds ratios and their 95% confidence intervals once the variables were identified using a p-value of 0.05 as a risk factor of statistical significance. The prevalence of tungiasis among children 5–14 years of age in Mettu rural woreda was 52 percent (n = 821). As a risk factor, large family size (AOR: 2.9, 95% CI: 2.13, 4.40); school attendance (AOR: 1.5, 95% CI: 1.02, 2.18), floor inside the house (AOR: 3.8, 95% CI: 1.76, 8.43); having sleeping bed (AOR: 0.16, 95% CI: 0.03, 0.82); access to protected water sources (AOR: 0.24, 95% CI: 0.15, 0.39); access to improved toilet facilities(AOR: 0.63: 95% CI: 0.44, 0.89); access to electric services (AOR: 0.30, 95% CI: 0.15, 0.62); and lack of own farmland (AOR: 0.36, 95% CI: 0.26, 0.50) were found. Therefore, planning and implementation of interventions focus on those risk factors that are particularly important. Water, sanitation and hygiene interventions, and livelihood improvement interventions are required to solve the problem in the setting.

## Introduction

Tungiasis is an ectoparasitic infestation caused by tunga penetrans infestation. The disease is prevalent in the Caribbean, South America, Africa, and India, particularly during the hot, dry season [1, 2]. Tungiasis is also a zoonotic disease that raises the burden of human parasites, resulting in considerable disabling morbidity in endemic areas [3, 4]. The periungual region of the toes is the most preferred site for the flea, although infestation can also occur in hands, elbows, and genital and anal regions [5]. The infestation is more common in children, particularly those aged 5–10 years [6], living with reservoir domestic animals like cats, dogs, and pigs [7], poor personal hygiene, poor sanitation of the housing and residential environment, and a lack of foot wear are all risk factors for tungiasis infestation [8, 9].

There is a lack of information on the global occurrence of tungiasis. However, 88 countries are thought to be infested with jigger, according to global estimates. The ecto-parasite is present in the Sub-Saharan region of Africa, including Sierra Leone, Ivory Coast, Nigeria, and Ethiopia, as well as South Africa; it is also found in Zanzibar and Madagascar [10]. According to a study conducted in Logos state, Nigeria, the prevalence of *Tunga penetrans* is 22.5 to 45.2% [11, 12]. A similar study from Tanzania, shows the prevalence of Tungiasis is 39% while in Uganda the prevalence was at 47% in children between 3–8 years of age [13]. A study conducted in a rural district of Rwanda shows that the prevalence of *Tungiasis* infection among children was 23%.

According to a study conducted in a rural community in Nigeria, having a sand or clay floor inside the home, as well as a traditional resting place outside the building, are significant risk factors for getting jiggers [9]. Sleeping on a non-solid floor also increases the risk of penetration [14]. A study from Brazil shows walking barefoot and the presence of garbage littering in the area increase the risk of infection [12, 15]. Lack of education and information about the circumstances were frequently listed as risk factors for having jiggers in the literature [16, 17]. Animal ponds, inadequate sanitation, and a lack of infrastructure are also major contributors to the high prevalence. Poor neighborhoods are frequently clustered outside of major cities, where there is more wildlife [17]. The shortage of waste disposal among vulnerable residents is becoming more apparent. Inadequate or non-existent sanitation [15, 18], a lack of clean water and soap, and the resulting poor sanitation are all major contributors to jiggers infestation [11].

According to studies conducted in Ethiopia, the prevalence of tungiasis ranged from 15 to 59 percent. According, the prevalence of tungiasis among children aged 5 to 14 years is 15 percent in Jimma, and 24 percent in Wolayita Sodo [19], while it is 58 percent in the Wensho district [8]. Tungiasis prevalence is influenced by socio-demographic factors such as mothers’ education, cat-owning families, wearing footwear rarely or never [8], poverty wages, poor housing and living conditions, and poor health seeking behavior [12]. However, data on the prevalence of tungiasis in children and its risk factors were limited to tack action in this particular area. Therefore, this study is aimed at investigating the prevalence of tungiasis and its risk factors among children in Mettu rural woreda of Ilu Abba Bor Zone, southwest Ethiopia.

The study’s findings assist health-care administrators at all levels, especially those looking at the population in the woreda, in understanding the scope of the issue. It also increases the ability to search out new alternative healthcare options in view of the community’s prevalence of tungiasis and risk factors. Furthermore, it leads to a better understanding of the risk factors associated with the prevalence of tungiasis in children aged 5 to 14 in the area.

## Methods and materials

### Study setting

This community-based cross-sectional study was conducted in Mettu Rural District, Ilu Aba Bor Zone, and Oromia Regional State, located in southwest Ethiopia, in October 2021. Mettu Rural District is one of the 14 districts of the Ilu Aba Bor Zone, located 620 km to the south-west of Addis Ababa. There are 30 kebeles in the Mettu rural district. Mettu Rural has a total population of 84,838, of which 42,334 are male and 42,504 are female (as of 2017) [20].

### Sample size determination and procedures

The sample size was estimated using the formula for single population proportion [21], using 95% confidence level, 0.05 margin of error and 58.7% population proportion. The final sample size was 821 based on the assumptions: 58.7% proportion of children 5–14 years taken from a study conducted in Ethiopia, which is similar to the current study area [22], 10% non-response rate, and a design effect of two.

The study participants were selected using multistage sampling of two stages. Accordingly, at the first stage, 9 kebeles (30% of the total kebeles) were selected randomly from the 30 kebeles using a lottery method. Following the selection of kebeles, the second stage involved the identification and registration of households with children aged 5–14 years in each selected kebeles prior to the study in order to obtain the sampling frame. Households were selected using a systematic sampling method using the sampling frame. Proportional allocation was used to determine the number of households from selected kebeles. Accordingly, Berowi-Shonkora (90; 982), Medalu (92; 1003), Tulube (80, 873), Sedo (98; 1069), Siba (96; 1047), Kachi (91; 993), Burusa (93; 1015), Kodo-Hiri (87; 949), and Geba-Guda (94; 1026) were the kebeles included, with their sample and target population S1 File. Where a household has more than one child who meets the criteria, only one child was included in the study by random selection. The data collection was done through physical examination of the children, interview of parents/guardians of the children, and observation of the home environment of the children.

### Data collection tools and procedures

Nurses collect data after receiving training on data collection instruments and procedures. The information was obtained via the following methods: physical assessment of the children, interviews with the children’s parents/guardians, and assessment of the children’s home environment. The children were examined physically for the existence of embedded tungiasis in their legs, feet, hands, and arms [8]. The children’s parents/guardians were interviewed using a standardized questionnaire that included demographic, socioeconomic, environmental, and behavioral factors, as well as disease-related conditions. The sanitation and other relevant conditions of the housing area were observed and assessed using a structured checklist.

The data collection tool was developed in English and translated to Afan Oromo S2 and S3 Files, and pre-tested on 5% of the study participants who were recruited from a similar setting in Hurumu District near Kebeles. The procedures and tools’ acceptability and applicability were validated during the pre–test. The respective supervisors reviewed all questioners on a daily basis for completeness, accuracy, and validity. The investigator monitored the overall data management operations.

Physical examination of jigger infestation characteristics found using the Fortaleza classification: from a dark and scraping spot in the skin to distinct craters like sores in the skin or supportive lesions in the natural history of illness [8]. Tungiasis was described as the presence of T. penetrans in the skin of any child 5–14 years of age chosen from the household members at the time of data collection, and any infestation case reports in the previous three months were considered positive for the disease in this study.

#### Data processing and analysis

The data was entered using Epidata version 3.1 [23] and analyzed using IBM SPSS version 20 [24]. The basic characteristics of the studied children and the prevalence of tungiasis were determined using frequency and percentage. We considered the risk of tungiasis (exposure of tungiasis infestation) as an outcome variable used bi-variable analysis to examine the risk factors for tungiasis exposure among children aged 5–14 years. The risk factors for *tungiasis* were identified using logistic regression. Those variables with a p-value less than 0.025 in the crude analysis were considered potential for multivariable analysis. The Hosmer-Lemeshow goodness-of-fit test statistic was performed for model adequacy. To assess the significance of the association, the adjusted odds ratio (AOR) with its corresponding 95% confidence interval (CI) was used, and variables with a p-value of 0.05 were considered to have a statistically significant association between risk factors and tungiasis infestations.

### Ethical considerations

This study was approved by the Mettu University’s Ethical Approval Committee. Written informed consent was obtained from each participant after providing full information about the study’s goals, selection criteria, confidentiality, and benefits. Participants were informed of their right to refuse or withdraw from the study at any time. The parents/guardians provided written informed consent before each interview. The positive cases of tungiasis were referred to nearby health facilities, as well as health advice for parents/guardians.

## Results

### Socio-demographic characteristics

The socio-demographic characteristics of study participants were presented in table one below (Table 1). Accordingly, a total of 821 respondents participated in this study with a response rate of 100%. Of the 821 respondents, 520 (63.3%) were females. The majority of the study participants were 637 (77.6%) were school attending students. About 354 (43.5%) of mothers can read and write, 173 (21.1%) cannot read and write, 215 (26.5%) attended some primary education, and only 215 (9.6%) attended secondary and above education.

**Table 1:** A socio-demographic characteristic of the respondent’s on the study of prevalence and risk factors of *Tungiasis* among children 5–14 years in Mettu rural woreda, 2020.

Variables	Categories	Frequency	Percentage
**Sex of child (n = 821)**	Female	520	63.3
	Male	301	36.7
**School attendance (n = 821)**	Not attending	184	22.4
	Attending	637	77.6
**Religion (n = 821)**	Orthodox	285	34.7
	Protestant	241	29.4
	Muslim	291	35.4
	Catholic	4	0.5
**Mother Education (n = 821)**	Cannot read and write	173	21.1
	Read and write	354	43.1
	Primary	215	26.2
	Secondary and above	79	9.6
**Father education (n = 805)**	Cannot read and write	30	3.7
	Read and write	487	60.5
	Primary	188	23.4
	Secondary and above	100	12.4
**Mother’s occupation (n = 821)**	Farmer	139	16.9
	Housewife	474	57.7
	Government employee	45	5.5
	Merchant	21	2.6
	Student	126	15.3
	Daily laborer	16	1.9
**Father’s occupation(n = 805)**	Farmer	565	70.2
	Government employee	21	2.6
	Merchant	75	9.3
	Student	7	0.9
	Daily laborer	133	16.5
	Other	4	0.5
**Family size (n = 821)**	Less than five	407	49.6
	Five and above	414	50.4
**Age category (n = 821)**	5–9 Years	429	52.3
	10–14 Years	392	47.7
**Main source of family income(n = 821)**	Farming	547	66.6
	Livestock	26	3.2
	Salary	82	10
	Petty trading	2	0.2
	Daily laborer	149	18.1
	Hand craft	15	1.8

In terms of father education, approximately 487 (60.5%) can read and write, 30 (3.7%) are unable to read and write, 188 (23.4%) have some primary education, and 100 (12.4%) have some secondary and higher education. Concerning family occupation, about 474 (57.7%) of mothers were engaged as home mothers as their main daily work, 565 (70.2%) of family heads were engaged in farming, and the rest were occupied with market work, daily labor and government employees. The main family size was five members, about half of 407 (49.6%) households had less than five family members, and 414 (50.4%) were with above five family size. The children’s ages ranged from 5 to 9 years for 429 (52.3%) and 10 to 14 years for the remaining 392 (47.2%) participants. The mean age was 10.18 years, and a standard deviation of 2.66 years.

#### Housing condition

The housing condition and sanitation status of the households were presented in Table 2 below (Table 2). In this study, more than half of 553 (67.4%) had a separate kitchen for cooking in their home. The majority of the households 664 (80.9%) had access to improved water sources, about 194 (23.6%) had access to improved toilet facilities, only 50 (6.1%) had access to improved indoor floor (i.e cemented floor), 709 (86.4%) had roof cover of iron sheet, and 811 (98.8%) wood and mud wall type. Of the total participants, about 524 (63.8%) households own a plot of farm land, 487 (59.3%) had at least one life stock and 283 (34.5%) reported the presence of other domestic animals in their compounds. The majority of the children, 764 (93.1%) reported they had shoes, and 746 (90.9%) practiced bathing at least twice per week.

**Table 2:** Housing condition of the respondents participated in study of prevalence and risk factors of *tungiasis* among children 5–14 years in Mettu rural woreda, 2020.

Variables	Categories	Frequency	Percentage
**Separated kitchen(n = 821)**	Yes	553	67.4
	No	268	32.6
**Water sources used by households (n = 821)**	Pipe water (Protected)	97	11.8
	Protected well	314	38.2
	Protected spring	253	30.8
	Unprotected well	33	4
	Unprotected spring	54	6.6
	Surface water(unprotected)	70	8.5
**Toilet facilities (n = 821)**	Ventilated improved toilet	30	3.7
	Pit latrine with slab (improved)	164	20
	Pit latrine without slab (unimproved)	607	73.9
	Open field/no facility	20	2.4
**Have livestock (n = 821)**	No	334	40.7
	Yes	487	59.3
**House floor (n = 821)**	Cemented/ ‘liishoo’	771	93.9
	Earth and Mud	50	6.1
**House roofing (n = 821)**	Leaf/grass	112	13.6
	Iron sheet	709	86.4
**House walls type (n = 821)**	Wood and mud	811	98.8
	Wood sticks	10	1.2
**Have electric service (n = 821)**	Yes	59	7.2
	No	762	92.8
**Have Radio (n = 821)**	Yes	108	13.2
	No	713	86.8
**Have sleeping bed (n = 821)**	Yes	12	1.5
	No	809	98.5
**Own farm land (n = 821)**	Yes	524	63.8
	No	297	36.2
**Frequency of bathing (n = 821)**	Daily	43	5.2
	Twice per day	746	90.9
	3 times a day	32	3.9
**Use shoes(n = 821)**	Yes	764	93.1
	No	57	6.9
**Type of shoes(n = 764)**	Open shoes	388	50.8
	Closed shoes	376	49.2
**Presence of domestic animal in their compound (n = 821)**	Yes	283	34.5
	No	538	65.5
**Frequency of floor cleaning (n = 821)**	Daily	66	8
	Twice a day	659	80.3
	Three times a day	8	1
	Once a week	88	10.7

### Prevalence of *tungiasis* among children

Table 3 shows the prevalence of tungiasis among children aged 5 to 14 in Mettu rural woreda (Table 3). Accordingly, the prevalence of *tungiasis* among 5–14 years of children the study area was 52.3% (48.8%–55.7%). Of the total respondents only 69 (8.4%) agree that a tungiasis is as a public health disease, and 221 (26.9%) reported the community discriminate the person with *tungiasis* infestation. The majority of the respondents 746 (91.1%) argue parasite is the main cause the disease, and 59 (7.2%) reported it caused because of dirty environment. More than half 632 (77%) reported as the disease is not a public concern and not a point of discussion at family level. Skin infection is the major complication of the disease reported from 791 (96.3%). Of those reported at least one exposure the case 414 (96.1%) sagest the own removal of the flea larvae as solution for home remedy. The prevention methods were 614 (74.8%) personal hygiene, using chemicals 76 (9.3%), using shoes 6 (0.7%), and early removal of the larvae 125 (15.2%). Of the total respondents, 198 (24.1%) reported tungiasis is a seasonal in which winter season is a pick season 171 (86.4%) for the infestation in the study area.

**Table 3:** Prevalence of *Tungiasis* among children 5–14 years in Mettu rural woreda, 2020.

Variables	Categories	Frequency	Percentage
**Any case of Tungiasis in the last three months(n = 821)**	Yes	429	52.3
	No	392	47.7
**Agree tungiasis is disease (n = 821)**	Yes	69	8.4
	No	752	91.6
**Person with Tungiasis infestation isolated /discriminated in the community (n = 821)**	Yes	221	26.9
	No	600	73.1
**Major cause of Tungiasis(n = 821)**	Parasite	746	91.1
	Whichcraft	12	1.5
	Cult	2	0.2
	Worms	2	0.2
	Dirty	59	7.2
**Discuss about *Tungiasis* with family**	Yes	189	23
	No	632	77.
**Measure taken when occur (429)**	Own surgery or remove	414	96.5
	Visit health facility	11	2.6
	Ignore	4	0.9
**Any disease related / complication to that (n = 821)**	Tetanus	19	2.3
	Skin infection	791	96.3
	Disability	11	1.4
***Tungiasis* is seasonal disease (n = 821)**	Yes	198	24.1
	No	623	75.9
**Season of high infestation(n = 198)**	Autumn	27	13.6
	Winter	171	86.4
**Prevention methods(n = 821)**	Use chemical	76	9.3
	Personal hygiene	614	74.8
	Shoes	6	0.7
	Remove	125	15.2

### Risk factors of *tungiasis* among children

In this analysis, many variables were explored to test the association of adjustment of variables using logistic regression to predict variables that were associated with exposure of tungiasis during the crude analysis. In the final model, family size, school attendance, indoor house floor type, access to sleeping bed, family access to water supply, family access to toilet facility, family access to electric service, and farm land ownership were risk factors for tungiasis infestation in children aged 5 years to 14 years (Table 4). Accordingly, family size (AOR/ 95% CI:2.93/2.133–4.402), school attending (AOR/95% CI:1.49/1.017–2.186), indoor floor type (AOR/95% CI: 3.851/1.759–8.43), having sleeping bed(AOR/95% CI:0.16/0.03–0.82), access to protected water sources(AOR/95% CI: 0.245/0.15–0.39), access to improved toilet facilities (AOR/95% CI: 0.628/0.44–0.89), access to electric services (AOR/95% CI:0.305/0.15–0.62), and having own farmland (AOR/95% CI: 0.365/0.26–0.50).

**Table 4:** Multivariable analysis of risk factors of tungiasis among children 5–14 years in Mettu rural woreda, southwest, Ethiopia.

Variables	Categories	Case	No case	Crude Odds Ratio			Adjusted Odds Ratio		
				COR	CI	P-value	AOR	CI	P-value
**Family size**	Less 5	171	236	1			1		
	5 and above	258	156	2.28	1.7, 3.0	<0.0001	2.9	2.13, 4.04	0.005**
**Have Bed**	No	427	382	1			1		
	Yes	2	10	0.18	0.02, 0.82	0.02	0.16	0.03, 0.83	0.029*
**School Attending**	No	82	102	1			1		
	Yes	347	290	1.49	1.1, 2.1	0.018	1.5	1.02, 2.19	0.041*
**Floor type**	Cemented	391	380	1			1		
	Earth and mud	38	12	3.08	1.59, 5.9	0.001	3.85	1.76, 8.43	0.001**
**Water Source**	Unprotected	124	33	1			1		
	Protected	305	359	0.23	0.15, 0.34	<0.0001	0.25	0.15, 0.39	<0.001**
**Toilet Facilities**	Unimproved	353	274	1			1		
	Improved	76	118	0.5	0.36, 0.69	<0.0001	0.63	0.44, 0.90	0.010**
**Electric**	No	418	344	1			1		
	Yes	11	48	0.19	0.096, 0.37	<0.001	0.31	0.15, 0.62	0.001**
**Radio**	No	360	353	1			1		
	Yes	69	39	1.73	1.14, 2.63	0.01	1.13	0.69, 1.86	0.621
**Land ownership**	No	326	198	1			1		
	Yes	103	194	0.34	0.24, 0.43	<0.005	0.36	0.26, 0.51	<0.001**

Key:

** significant at P- value <0.025

* Significant at P-value <0.05.

## Discussion

This study demonstrated the prevalence of tungiasis and its risk factors among children aged 5 years to 14 years in Mettu rural woreda. The prevalence of tungiasis among children 5–14 years of age in Mettu rural woreda was 52.3%. This finding is higher than similar studies in Ethiopia in Wolayita Sodo 23.9% and Jimma 15.15% [25], Nigeria 45.2% [12], Uganda 22.5% [26], and lower than the study conducted in Densho district in Ethiopia 58% [8].

Of the total respondents, only 69 (8.4%) agree that tungiasis is a public health disease, and 221 (26.9%) reported the community discriminates against people with *tungiasis* infestation. More than half of 632 (77%) reported the disease is not a public concern and not a point of discussion at the family level. This demonstrates that the disease is widely discriminated against and ignored, even at the community level. The majority of the respondents 746 (91.1%) argue parasites are the main cause of the disease, and 59 (7.2%) reported it was caused because of the dirty environment.

Skin infection is the major complication of the disease reported by 791 (96.3%) respondents. The prevention methods were 614 (74.8%) personal hygiene, using chemicals 76 (9.3%), using shoes 6 (0.7%), and early removal of the larvae 125 (15.2%). Tungiasis is a seasonal disease, with the winter season being a pick season, according to 198 (24.1%) of the total respondents, and 171 (86.4%) for the infestation in the study area. Of those who reported exposure to the case, 414 (96.1%) suggest the own removal of the flea larvae as a solution for a home remedy. As a result of self-removal, which is more common in children, and the use of unsanitary tools, other fleas can embed in the skin, potentially leading to secondary infection as well as permanent disability.

The risk factors for tungiasis exposure among children aged 5 to 14 years were family size, school attendance, indoor house floor type, access to sleeping bed, family access to water supply, family access to toilet facilities, family access to electric service, and farmland ownership (Table 4). In this study, children from large families (those with five or more siblings) were 2.9 times more likely to contract tungiasis than children from small families (AOR/95% CI:2.93/2.133–4.402). Large family sizes in the study area may increase the exposure because each child may have a play station that the probability of getting the fleas increases. Similarly, child care in large families in rural Ethiopia is insufficient, resulting in high exposure to such neglected diseases. A study from northern Ethiopia suggests that less child care among rural Ethiopian communities increases the risk of stunting, with large family sizes being at a higher risk of less child care [27]. The stunted child is more vulnerable to many health risks, including tungiasis.

Children attending school were 1.49 times more likely risk than not attending children (AOR/95% CI:1.49/1.017–2.186). This discovery demonstrates that the school environment has a dusty floor, which increases the habitat of fleas. In Ethiopia, less school health intervention is in place, especially in rural areas where class rooms are full of dust where students attend learning. Children from households having mud and earth house floors were 3.8 times more likely risk than other types of indoor cemented floor (AOR/95% CI: 3.851/1.759–8.43). Similarly, floor types of mud and earth are more favorable for the growth of fleas. This finding is consistent with study results from Uganda [12].

Housing conditions and hygiene practice at family level are very important in the control of many communicable diseases. This study result shows children whose family had a sleeping bed were 84% less risk than those lacking a sleeping bed (AOR/95% CI:0.16/0.03–0.82). Having a sleeping bed reduces the risk of exposure because fleas grow in dust. Similarly, children with family access to protected water sources were 75.5% less risk than those lacking water improved water sources (AOR/95% CI: 0.245/0.15–0.39). This finding is similar to the result from Kenyan [22]. Availability of water supply facilitates hygiene practices, in which access to water initiates the children and family to wash their bodies, clothes, and clean their indoor environment.

In the current study, families with access to improved toilet facilities were 37% less likely risk than those lacking access to toilet facility access (AOR/95% CI: 0.628/0.44–0.89). The children from families with access to electric services were 69.5% less risk than those lacking electric service (AOR/95% CI:0.305/0.15–0.62). The children from families that own farm land were 63.5% less risk than those who own farm land (AOR/95% CI: 0.365/0.26–0.50). Access to toilet facilities, electricity, and one’s own farmland are indicators of a high-class economy in a rural community, and having such amenities improves one’s standard of living. In any community, having a good lifestyle is conventionally the way to reduce communicable disease. As a result, it has a similar effect on reducing tungiasis infestation in the study area. This finding is consistent with the study results from Densho district in Ethiopia [12]. However, this study did not address other age segments of the population that may have high or less prevalence even in the same family member. The risk among children may not be the same as the risk among the rest of the population in the same kebeles.

## Conclusion

This study found a high prevalence of tungiasis infestation among children aged 5 to 14 in Mettu Woreda. Family size, school attendance, indoor house floor type, access to sleeping bed, family access to water supply, family access to toilet facilities, family access to electric utility, and farm land ownership were all statistically significant risk factors in the study area. Therefore, planning and implementing interventions for school health services to reduce the risk of infestation. Because this untreated disease poses a high risk to schoolchildren and is likely to increase absenteeism, school sanitation and hygiene promotion should be integrated and implemented. Community health workers must educate households and caregivers on the importance of not ignoring this common illness that causes disability. Finally, this study used a cross-sectional survey to assess risk and prevalence and was unable to establish a cause and effect relationship, a longitudinal study, particularly an interventional study, is recommended for future research.

## Supporting information

S1 File Mettu rural woreda population in 2020 G.C. (PDF)

S2 File Questionnaire (English version). (PDF)

S3 File Questionnaire (Afan Oromo version). (PDF)

S4 File (SAV)
