# Supplementary material for: Prevalence of Tungiasis and its risk factors of among children of Mettu woreda, southwest Ethiopia, 2020
Source: PLoS One. 2022 Jan 5;17(1):e0262168. doi: 10.1371/journal.pone.0262168 (PMC8730454; doi:10.1371/journal.pone.0262168)
Supplement: S1 File — (PDF) [file pone.0262168.s001.pdf]

S 1: Mettu Rural Woreda population in 2020 G.C (Source: Mettu Rural Woreda health Office)

S/N	Name kebeles	Male	Female	Total population	Households	5 to 14yrs population	Target	Sample
1	Adale-Gumer	2033	2033	4066	847	1268		
2	Adale-Haro	1177	1176	2353	490	734		
3	Adale-Kaso	986	986	1972	411	615		
4	Adelle-Bise	2652	2652	5304	1105	1654		
5	Agelo-Eko	1239	1239	2478	516	773		
6	Agelo-Warkyi	1233	1232	2465	514	769		
7	Alga-Gossuu	530	529	1059	221	330		
8	Alga-Guracha	688	687	1375	286	429		
9	Alle-buya	2206	2205	4411	919	1375		
10	Berowi-Gabisa	1078	1078	2156	449	672		
11	Berowi-Shonkora	990	989	1979	412	982	982	90
12	Boto	1250	1249	2499	521	779		
13	Burusa	1579	1578	3157	658	1003	1,003	92
14	Gaba-Guda	1622	1621	3243	676	873	873	80
15	Gayi	931	931	1862	388	581		
16	Huma-Didu	2186	2186	4372	911	1363		
17	Kawo-Chatu	1005	1005	2010	419	627		
18	Kawo-Chogi	1172	1172	2344	488	731		
19	Kechi	785	785	1570	327	1069	1,069	98
20	Kemise	2846	2846	5692	1186	1775		
21	Kodo-Hiri	2684	2684	5368	1118	1047	1,047	96
22	Made	1595	1594	3189	664	994		
23	Medalu	808	808	1616	337	993	993	91
24	Sardo	1472	1471	2943	613	918		
25	Sedo	1569	1569	3138	654	1015	1,015	93
26	Siba	1410	1409	2819	587	949	949	87
27	Tobecha	851	874	1725	359	538		
28	Tulubbe	2921	2920	5841	1217	1026	1,026	94
29	Warkayi-Dire	1475	1475	2950	615	920		
30	Wuchi	906	906	1812	378	565		
**Total**		**43,892**	**43,879**	**87,771**	**18,286**	**27,367**	**8,958**	**821**

Source: Mettu Rural woreda health Office

Printed by BoltPDF (c) NCH Software. Free for non-commercial use only.
