# Supplementary material for: Prevalence of Tungiasis and its risk factors of among children of Mettu woreda, southwest Ethiopia, 2020
Source: PLoS One. 2022 Jan 5;17(1):e0262168. doi: 10.1371/journal.pone.0262168 (PMC8730454; doi:10.1371/journal.pone.0262168)
Supplement: S2 File — (PDF) [file pone.0262168.s002.pdf]

S2: Questionnaire (English version)

Participant information sheet

Good morning/Good afternoon? My name is ___________________. I am working as a data collector for the study being conducted in this kebele by a research team from Mettu University, College of Health Science, and the D   epartment of Public Health.

I would appreciate it if you would give me your time to explain the study and how you were chos en as a study participant.

- **The study title:** Prevalence of Tungiasis and its' risk factors among children 5-14 years in Mettu rural woreda ,in Ilu Aba Bor Zone, Southwest Ethiopia
- **Purpose of the study :** The study's findings are critical for the District and the Zonal Health Department in planning an intervention program to prevent tungia   sis among children aged 5–14 years in that area by improving child health in general.
- **Procedure and duration:** I will be interviewing you using a questionnaire to provide me with pertinent data that is helpful for the study. The interview will take 30 minut es. I will visit your house, toilet, and waste disposal points. I will see one child aged 5 to 14 years old. I may also ask you some questions related to the hygiene practices of your family members. So I kindly request that you spare me this time. This su   rvey will assist the government in developing programs to reduce the problems of tungiasis among children aged 5–14 years.
- **Risks and benefits :** The risk of participating in this study is minimal and takes a few minutes of your time. There would not be any d   irect payment for participating in this study. But the findings from this research may reveal important information for health facilities and local health planners.

Printed by BoltPDF (c) NCH Software. Free for non-commercial use only.

- **Confidentiality :** Any information you provide us with will be confidential. There will be no information that will identify you in particular. The findings of the study will be general for the study population and will not reflect anything particular about individual people or housing. The questionnaire will be coded to exclude names. No reference will be made in oral or written reports that could link participants to the research.
- **Rights :** Participation for this study is fully voluntary. You have the right to declare to participate or not in this study. If you decide to participate, you have the right to withdraw from the study at any time and this will not label you for any loss of benefits which you otherwise are entitled. You do not have to answer any question that you do not want to answer.
- **Contact address :** If you have any questions or inquiries any time about the study or the procedure please contact, Mr. Sime Daba, at mobile Phone: +251 -911541940 or Dereje Oljira +251 -917818339

Declaration of informed voluntary consent

I have read or had read to me the participant information sheet. I have clearly understood the purpose of the research, the procedures, the risks and benefits, issues of confidentiality, the right to participate and the contact address for any queries. I was informed that I have the right to withdraw from the study at any time or not answer any question that I do not want. Therefore, I declare my voluntary consent to participate in this study with my initials (signatures) as indicated below. Signature of the participant/care giver/ parent ______________

Signature of the data collector _______________ date___________________________

Kebele Code____________ Participant code________

Printed by BoltPDF (c) NCH Software. Free for non-commercial use only.

Questionnaire

Introduction : Thank you for your participation. To begin with, let me ask you about the general information about your family.

A. Socio -demographic Characteristics of the respondent				
**S.N**	**Questions**	**Response**	**Code**	**Skip**
SD01	Are you the family head of this household?	Yes	[ ] 1	
		No	[ ] 2	
SD02	How old are you?	___________________years		
SD03	How many people usually live with you in your house? (family size)	_______________________		
SD04	What is the father's educational level?	Cannot read and write	[ ] 1	
		Read and write	[ ] 2	
		Primary	[ ] 3	
		Secondary and above	[ ] 4	
SD05	What is the wife's education al level?	Cannot read and write	[ ] 1	
		Read and write	[ ] 2	
		Primary	[ ] 3	
		Secondary and above	[ ] 4	
SD06	What is the main occupation of the father?	Farmer	[ ] 1	
		House wife	[ ] 2	
		Employee (Gov't)	[ ] 3	
		Merchant	[ ] 4	
		Student	[ ] 5	
		Daily laboror	[ ] 6	
		Others (Specify)_________	[ ] 7	
SD07	What is the main occupation of the wife?	Farmer	[ ] 1	
		House wife	[ ] 2	
		Employee (Gov't)	[ ] 3	
		Merchant	[ ] 4	
		Student	[ ] 5	
		Daily laboror	[ ] 6	
		Others (Specify)_________	[ ] 7	
SD08	What is your religion?	Orthodox	[ ] 1	
		Protestant	[ ] 2	
		Muslim	[ ] 3	
		Catholic	[ ] 4	
		Other, specify ___________	[ ] 5	
SD09	What is the main source of your family income? *Petty trading: (including sale fire - wood, charcoal, grass, localbrewery)*	Farming	[ ] 1	
		Salary	[ ] 2	
		Petty trading	[ ] 3	
		Daily laborer	[ ] 4	
		Hand craft/ artisan	[ ] 5	

Printed by BoltPDF (c) NCH Software. Free for non-commercial use only.

		remittances		
		Other, specify ___________	[ ] 6	

B. Households' housing conditions and sanitation status

S.N	Questions	Response		Coding	Skip
**HC01**	What is the main source of water for members of your household?	Piped water		[ ] **1**	
		Protected Dug well		[ ] **2**	
		Unprotected dug well		[ ] **3**	
		Protected spring		[ ] **4**	
		Unprotected spring		[ ] **5**	
		Rainwater		[ ] **6**	
		Tanker truck		[ ] **7**	
		Surface water (River, pond)		[ ] **8**	
		Bottled water		[ ] **9**	
		Others__________________		[ ] **10**	
**HC02**	What kind of toilet facility does your household own?	Flush or pour flush toilet		[ ] **1**	
		Ventilated improved pit latrine		[ ] **2**	
		Pit latrine with slab		[ ] **3**	
		Pit latrine without slab/ open pit		[ ] **4**	
		No facility/bush/field		[ ] **5**	
		Others (specify _____________		[ ] **6**	
**HC03**	Does your household have:<br>*More than one answer is possible*	Electric utility access?	**Yes**	[ ] **1**	
			No	[ ] **2**	
		A watch?	**Yes**	[ ] **1**	
			No	[ ] **2**	
		A radio?	**Yes**	[ ] **1**	
			No	[ ] **2**	
		A television?	**Yes**	[ ] **1**	
			No	[ ] **2**	
		A mobile telephone?	**Yes**	[ ] **1**	
			No	[ ] **2**	
		A non -mobile telephone?	**Yes**	[ ] **1**	
			No	[ ] **2**	
		A refrigerator?	**Yes**	[ ] **1**	
			No	[ ] **2**	
		A table?	**Yes**	[ ] **1**	
			No	[ ] **2**	
		A chair?	**Yes**	[ ] **1**	
			No	[ ] **2**	
		A bed?	**Yes**	[ ] **1**	
			No	[ ] **2**	
		An electric mitad?	**Yes**	[ ] **1**	
			No	[ ] **2**	

Printed by BoltPDF (c) NCH Software. Free for non-commercial use only.

**HC0 4**	Do you have separate rooms that are used as kitchens? (Observe and tick)	Yes	[ ] **1**	
		No	[ ] **2**	
**HC0 5**	What is the main living house's floor? (observation) *Tick ONLY ONE answer*	Earth and mud	[ ] **1**	
		Wooden	[ ] **2**	
		Ceramic tiles	[ ] **3**	
		Cement/bricks	[ ] **4**	
		Other [specify]____________	[ ] **5**	
**HC0 6**	What is the main living house's roof? (observation) *Tick ONLY ONE answer*	Grass /leaf	[ ] **1**	
		Plastic sheets	[ ] **2**	
		Wood	[ ] **3**	
		Iron sheet	[ ] **4**	
		Cement	[ ] **5**	
		Other [specify]_____ ______	[ ] **6**	
**HC0 7**	What is the main living house's wall? (observation) *Note: Tick ONLY ONE answer, if more than one used tick the one covers large space*	Wooden and mud	[ ] **1**	
		Wood sticks	[ ] **2**	
		Cement	[ ] **3**	
		Stone with lime/cement	[ ] **4**	
		Bricks	[ ] **5**	
		Wood plank/Shinles	[ ] **6**	
		Other [specify]______________	[ ] **7**	
**HC0 8**	Does this household own their own farm land that can be used for agriculture?	Yes	[ ] **1**	
		No	[ ] **2**	
**HC09**	Does this household own any livestock, herds, or farm animals?	Yes	[ ] **1**	
		No	[ ] **2**	
**HC10**	How many times do the children of this household usually take baths in a week?	Daily	[ ] **1**	
		Twice	[ ] **2**	
		Thrice	[ ] **3**	
		None	[ ] **4**	
		Others Specify…………………	[ ] **5**	
**HC11**	What is your compound ground covered with? (observe)	Grass	[ ] **1**	
		Soil	[ ] **2**	
		Both Grass and Soil	[ ] **3**	
		Others (specify) ……………..	[ ] **4**	
**HC12**	Do domestic animals live within the compound?	Yes	[ ] **1**	
		No	[ ] **2**	
**HC13**	How often do you clean the floor in your house in a week?	________________________ ____________________		
**HC14**	What is the common resting place in the homestead for members of your household?	Under The Tree	[ ] **1**	
		In the Class Room	[ ] **2**	
		Veranda	[ ] **3**	
		Road Paths	[ ] **4**	

Printed by BoltPDF (c) NCH Software. Free for non-commercial use only.

		Others (Specify) ……………	[ ] 5	

C. Knowledge of the respondents about Tungiasis and infestation

**KT01**	What is the major cause of this disease?	Parasite	[ ] **1**	
		Witchcraft	[ ] **2**	
		Cult	[ ] **3**	
		Mental illness	[ ] **4**	
		Worms	[ ] **5**	
		Dirt	[ ] **6**	
		Don't know	[ ] **7**	
		Others (Specify)…………….	[ ] **8**	
**KT02**	Have you ever discuss with your household members about Tungiasis?	Yes	[ ] **1**	
		No	[ ] **2**	
**KT03**	Do Tungiasis is a disease?	Yes	[ ] **1**	
		No	[ ] **2**	
**KT04**	How can tungiasis be managed/ prevented?	________________________________ ________________________________		
**KT05**	Has any household member 5 to 14 year age has been infested by Tungiasis in the last three months? **Note: Use clinical evaluation to tick this question. Look the selected child and tick the correct result** .	Yes	[ ] **1**	If yes give your advice and help the family.
		No	[ ] **2**	
**KT06**	Age of child	________in Year		
**KT07**	Sex of child	Female	[ ] **1**	
		Male	[ ] **2**	
**KT08**	Schooling of the child	Not attending school	[ ] **1**	
		Attending school	[ ] **2**	
**KT09**	How children wear shoes? (Observe, whether the child is using shoes during your observation)	Wearing Closed Shoes	[ ] **1**	
		Wearing Open Shoes	[ ] **2**	
		Walk Bear footed	[ ] **3**	
		Others (Specify)____	[ ] **4**	
**KT10**	What action do you take when infested?	________________________________ ____________________		
**KT11**	Are there other diseases/infections that are related to Tungiasis	Tetanus	[ ] **1**	
		Skin Infections	[ ] **2**	
		Disability	[ ] **3**	
		Others (specify) ………	[ ] **4**	
**KT12**	Is the infestation of tungiasis seasonal?	Yes	[ ] **1**	
		No	[ ] **2**	
**KT13**	If Yes, when is the infestation of tungiasis high?	________________________________ ________________________________		
**KT1 4**	Does the community isolate/ discriminate a person with tungiasis?	Yes	[ ] **1**	
		No	[ ] **2**	

Thank you your participation, this end of my questions!

Printed by BoltPDF (c) NCH Software. Free for non-commercial use only.
