# Supplementary material for: Prevalence of Tungiasis and its risk factors of among children of Mettu woreda, southwest Ethiopia, 2020
Source: PLoS One. 2022 Jan 5;17(1):e0262168. doi: 10.1371/journal.pone.0262168 (PMC8730454; doi:10.1371/journal.pone.0262168)
Supplement: S3 File — (PDF) [file pone.0262168.s003.pdf]

# Unkaa Odeefannoo

Akkami bultan/akkam ooltan?Ani maqaan koo____________________n jedhama. Ganda keessan kana kanan dhufeef, garee qorannoo fayyaa hawaasaa kan yuuniversiitii Mattuu koolleejjii saayinsii fayyaa, muummee fayyaa hawwasaa irra bakka bu'een. Ganda kana keessaa, akka carraa ta'ee qorannoo keenya keessatti akka hirmaattaniif ; filatamtaniituu. Waa'ee qorannichaa isiniif ibsaa, mee hayaataan na caqasaa.

- **Qorannoon keenya mata -dureen** isaa, ' ***Sakattoo tatamsai'ina Buujalee fi saaxilamummaa daa'immanni umuriin 5 hanga 14 giddi jirani irratti saba ba ta'uu malan , aana Mattuu baadiyya keessatti, godina Iluu Abbaa Boor, Kibba lixa ,Itoophiyaa***.
- **Galmi qorannichaa:** Bu'aa qorannoo kanarraa argamuun fayyadamuun dhaabbileen fayyaa fi dhimmam toonni fayyaa hawaasa a godin aa, aanaa fi hosipitaalonni dhibee muujalee kan a hirrisuun fayya d a'immanii fooyyessuuf, kaarfachuu fi mala dhahuuf akka isaan gargaaruuf yaadameetu.
- **Adeemsa qorannichaa fi yeroo:** Gaafilee haala qabiinsa manaa, haala maati i, qulqullinaa, fi dhimmo ota kanaaf nu gargaaran ilaaluun unkaa qophaa'e irratti guutuun ragaan funaanu un. Itti dabalataan ijoollee miseensa maatii ke essanii ta'anii , umuriin isaanii waggaa 5 hnaga 14 ta'an keessaa nama tokko miilla, harka isaanii irra ilaaluun mujaleef saaxilamuu isaani nan ilaala. Waanan argesi waraqaa kana irrattan galmeessa. Walumaa galatti yeroo hanga daqiiqaa 30 isin jalaa fudhachuu danda'a.
- **Miidhaa fi bu'aa:** Isin qorannoo kana irratt hirmaachuu keessanif, miidha isin irra ga'u tokkoyyuu hin jiru. Hirmaachuu keessaniifis kallattiin bu'aan maallaqaanis ta'ee faayidaan addaa hin jiru. Garuu immoo deebii keessaan irraa beekumsa argamuun fayyaa



Printed by BoltPDF (c) NCH Software. Free for non-commercial use only.

daa' immanii aanaa keessanii fooyyessuf dhibee kana dhabamsiisuu keessatti warra kaaroora fayya baasuuf raawwachiisu baay'ee fayyada.

- **Iccitii:** ragaan isin nuuf kennitan maqaa keessanii wajji n hin galmaa'u. Ragicha kan kenne isin ta'uu wanti ibsu waraqaa keenya irrattis ta'ee gama kamiinuu hin beekamu. Garuu bu'aan qorannichaa, akkuma walii galaatti aanaa kana keessatti rakko kanaatu jira jedhamee gabaasuuf oola. Ragich bifa qindaa'een icctiin isaa ni eegama. Unka mallattessitan kana irrattis mallattoo qofa kees san. Kunis fedhii keessan ibsuu, bu' uraalee qorannoo eeguuf kan itti fayyadamnuudha. Maqaa keessani barreessuun isin irraa hin eegamu.
- **Mirga:** Hirmaachuu fi ta'ee yeroo feetanitti, addaan kutuuf mirga guutuu qabdu. Gaaffiin isin deebisuu hin barbaanne yoo i sin mudates; dhiisuuf ykn irra darbuu mirgi keessan eegamaadha. Murtoo keessan yoo ta'e, deebii kamiinuu osoo hin kenni n addaan kuttanii deemuu dandeessu.
- **Odeeffanoo dabalataaf:** Obbo Si mee Dhaabaa lakkoofsa bilbilaa +251 -911541940 ykn Obbo Darajjee Olji rraa dubbisuu ni dandeessu.

## Waliigaltee Mirkaneessa Hubtanii fedhiin hirmaachuu

Odeeffannoo qornnichaa, kaayyoo, adeemsa, bu'aa fi miidhaa qorannichaa naaf ibsamee , naaf dubbifamee odeeffannoo barbaadamu kennuuf itti walii galeera. Ibsa naaf kenname irraas yeroo fedhetti gaafficha addaan kutuu, gaaffii naaf h in mijanne deebisuu dhiisuu, akkasumas iccitiin ragaa ani kennuus akka eegamu hubadheera. Kunis mirkaneessuuf fedhii kootin hirmaachuuf mallattoo koo akka armaan gaditti kaa'eera.

Mallattoo hirmaata/hi rmaattu/guddiftuu hrimaataa qorannoo___________________

Mallattoo nama ragaa kana funaanee____________ Guyyaa_________



Printed by BoltPDF (c) NCH Software. Free for non-commercial use only.

Koodii Gandaa____________ Koodii hirmaataa qorannoo______________

# Gaaffi  lee qorannoo

**Seensa** :Hirmaachuuf fedha qabaachuu kees saniif galatoomaa. Mee jalqabuuf, g aaffilee walii gal aa irraan jalqaba. Waa'ee maatii fi qe'ee irraan jalqaba.

| **A.** | **Haala hawwaasummaa ilaalchisee** | | | |
|---|---|---|---|---|
| **T/L** | **Gaaffilee** | **Deebii** | **Koodii** | **Ce'umsa** |
| SD01 | Isin geggeessaa maatii kanaatii? | Eeyyeen | [ ] 1 | |
| | | Lakkii | [ ] 2 | |
| SD02 | Umuriin keessan meeqa? | Waggaa _______________ | | |
| SD03 | Maatiin keessan nama meeqa? | _______________________ | | |
| SD04 | Abbaan warraa mana kanaa hangaa kutaa meeqa baratan? | Barreessu fi dubbisuu hin danda'u | [ ] 1 | |
| | | Barreessuu fi dubbisuu danda'a | [ ] 2 | |
| | | Sadarkaa 1ffaa | [ ] 3 | |
| | | Sadarkaa 2ffaa fi isaa oli | [ ] 4 | |
| SD05 | Haati warraa mana kanaa hangaa kutaa meeqa baratan? | Barreessu fi dubbisuu hin danda'u | [ ] 1 | |
| | | Barreessuu fi dubbisuu danda'a | [ ] 2 | |
| | | Sadarkaa 1ffaa | [ ] 3 | |
| | | Sadarkaa 2ffaa fi isaa oli | [ ] 4 | |
| SD06 | Hojiin abbaa warraa maali? | Qonnaan bulaa | [ ] 1 | |
| | | Soortuu maatii | [ ] 2 | |
| | | Hojjetaa Mootummaa | [ ] 3 | |
| | | Daldalaa/t tu | [ ] 4 | |
| | | Barataa/t tu | [ ] 5 | |
| | | Hojii guyyaa | [ ] 6 | |
| | | kan biroo (Ibsi)_________ | [ ] 7 | |
| SD07 | Hojiin haadha warraa maali? | Qonnaan bulaa | [ ] 1 | |
| | | Soortuu maatii | [ ] 2 | |
| | | Hojjetaa Mootummaa | [ ] 3 | |
| | | Daldalaa/ ttu | [ ] 4 | |
| | | Barataa/ ttu | [ ] 5 | |
| | | Hojii guyyaa | [ ] 6 | |
| SD08 | Amantaan keessan maali? | Ortodookisii | [ ] 1 | |
| | | Protestaantii | [ ] 2 | |
| | | Isilaama | [ ] 3 | |
| | | Kaatoolikii | [ ] 4 | |
| | | Kan biroo, Ibsi ___________ | [ ] 5 | |
| SD09 | Maddi galii maatii keessanii inni guddaan maali inni? | Qonnaa qutuun | [ ] 1 | |
| | | Miindaadhaan | [ ] 2 | |



Printed by BoltPDF (c) NCH Software. Free for non-commercial use only.

| | | | | |
|---|---|---|---|---|
| | | Daldala xixiqqaa | [ ] 3 | |
| | | Hojii guyya guyyaa | [ ] 4 | |
| | | Oogummaa harkaa | [ ] 5 | |
| | | kan biroo, ibsi ___________ | [ ] 6 | |

## B. Haala manaa fi gulqullinaa ilaalchisee

| T/L | Gaaffilee | Deebii | | Koodii | Ce'umsa |
|---|---|---|---|---|---|
| **HC01** | Maatiin keessan madda bishaanii eessaa fayyadamtuu? | Bishaan Ujummoon dhufu | | [ ] **1** | |
| | | Boolla kununfame | | [ ] **2** | |
| | | Boolla hin kununfamne | | [ ] **3** | |
| | | Burqituu kununfame | | [ ] **4** | |
| | | Burqituu hin kununfamne | | [ ] **5** | |
| | | Bishaan roobaa | | [ ] **6** | |
| | | Bishaan birkaaan dhihaatu | | [ ] **7** | |
| | | Bishaan lagaa ya'u/ciisu | | [ ] **8** | |
| | | Bishaan qaruuraa | | [ ] **9** | |
| | | Kan biro /ibsi____________ | | [ ] **10** | |
| **HC02** | Maatiin keessan booliif eessatti fayyadam tuu u? | Mana fincaanii ammayyaa | | [ ] **1** | |
| | | Boolla mana fincaanii qilleensaa baasu fooyya'aa (VIP) | | [ ] **2** | |
| | | Boolla irra buusa/golgaa qabu | | [ ] **3** | |
| | | Boolla golgaa hin qabne | | [ ] **4** | |
| | | Dirree/bosona | | [ ] **5** | |
| | | Kan biroo/ibsi____________ | | [ ] **6** | |
| **HC03** | Maatiin Keessan; | Ibsaa elektrikaa qabdu? | **Eeyyee** | [ ] **1** | |
| | | | Lakki | [ ] **2** | |
| | | Sa'aatii harkaa qabduu? | **Eeyyee** | [ ] **1** | |
| | | | Lakki | [ ] **2** | |
| | | Raadiyoonii qabduu? | **Eeyyee** | [ ] **1** | |
| | | | Lakki | [ ] **2** | |
| | | Televijiinii qabduu? | **Eeyyee** | [ ] **1** | |
| | | | Lakki | [ ] **2** | |
| | | Bilbila moobayilaa qabduu? | **Eeyyee** | [ ] **1** | |
| | | | Lakki | [ ] **2** | |
| | | Bilbilaa dhaabbataa qabdu? | **Eeyyee** | [ ] **1** | |
| | | | Lakki | [ ] **2** | |
| | | Firiijii qabduu? | **Eeyyee** | [ ] **1** | |
| | | | Lakki | [ ] **2** | |
| | | Minjaala /xarabeezaa qabduu? | **Eeyyee** | [ ] **1** | |
| | | | Lakki | [ ] **2** | |
| | | Barcuma taa'umsaa qabdu? | **Eeyyee** | [ ] **1** | |
| | | | Lakki | [ ] **2** | |
| | | Siree ciisichaa qabduu? | **Eeyyee** | [ ] **1** | |
| | | | Lakki | [ ] **2** | |



Printed by BoltPDF (c) NCH Software. Free for non-commercial use only.

| | | | | |
|---|---|---|---|---|
| | | Eel ee elektrikaa qabduu? **Eeyyee** | [ ] 1 | |
| | | Lakki | [ ] 2 | |
| **HC05** | Iddoo nyaata itti bilcheessan addatti qabduu?(ilaalii guuti) | Eeyyee | [ ] 1 | |
| | | Lakki | [ ] 2 | |
| **HC06** | Manni jireenyaa isaan lafa/' walaliin ' maliin ijaarame?(Ilaaliiguuti) | Biyyoo | [ ] 1 | |
| | | Mukaa | [ ] 2 | |
| | | Seeraamiki | [ ] 3 | |
| | | Simintoo/lishoo | [ ] 4 | |
| | | KB [ibsi]____________ | [ ] 5 | |
| **HC07** | Manni jireenyaa isaanii guutuun isaa maaliin hojjetame? (Ilaaluun guuti) | Chaffee/ Marga | [ ] 1 | |
| | | 'Pilaasitikii /sharaa' | [ ] 2 | |
| | | Muka | [ ] 3 | |
| | | Qorqorroo | [ ] 4 | |
| | | Simintoo | [ ] 5 | |
| | | kan Biroo[ibsi]____________ | [ ] 6 | |
| **HC08** | Manni jireenyaa isaanii dhaabdoon isaa maal irraa hojjetame? (Ilaaluun guuti) | Mukaa fi dhoqqee dhoobame | [ ] 1 | |
| | | M uka hin dhoobamne | [ ] 2 | |
| | | Simintoo | [ ] 3 | |
| | | Dhakaa fi simmintoo | [ ] 4 | |
| | | Xuubii | [ ] 5 | |
| | | Muka fi sharaa ykn kabaa | [ ] 6 | |
| | | Kan biroo [ibsi]______________ | [ ] 7 | |
| **HC09** | Maatiin keessan lafa qonnaa qabduu? | Eeyyeen | [ ] 1 | |
| | | Lakkii | [ ] 2 | |
| **HC11** | Maatiin keessan horii qe'ee kana akka loon, bushaayee, kot te- duudaa fi re'ee ni qabaa? | Eeyyeen | [ ] 1 | |
| | | Lakkii | [ ] 2 | |
| **HC13** | Daa'imman miseensi maatii keessanii irra -jireessaan torbanitti yeroo hangam hangamiitiin dhaqna dhiqatu? | Guyya -guyyaan | [ ] 1 | |
| | | Yeroo lama | [ ] 2 | |
| | | Yeroo sadii | [ ] 3 | |
| | | Hin beekamu | [ ] 4 | |
| | | Kan biro(ibsi)………. | [ ] 5 | |
| **HC15** | Iddoon mo oraa keessaa malii n huwwifame/bakka qe'ee? (Ilaaluun guuti) | Margaan huwwifame | [ ] 1 | |
| | | Biyyoodha | [ ] 2 | |
| | | Margaa fi biyyoo | [ ] 3 | |
| | | Kan biro(ibsi)………….. | [ ] 4 | |
| **HC16** | Horiin/ bineeldonni mooraa keessa ni jiraatuu ? | Eeyyeen | [ ] 1 | |
| | | Lakkii | [ ] 2 | |
| **HC18** | Torban keessatti, mana keessan keessa yeroo hanga hangamiin qulqulleessitu? | ________________________<br>_____________________ | | |
| **HC20** | Yeroo baayy'ee iddoo maatiin keessa n itti haara galfatu eessa? | Muka jala | [ ] 1 | |
| | | Kutaa manajireeyaa keessa | [ ] 2 | |
| | | Mana jala | [ ] 3 | |
| | | Daandii cinaa | [ ] 4 | |



Printed by BoltPDF (c) NCH Software. Free for non-commercial use only.

| | | | | |
|---|---|---|---|---|
| | | Kan biroo(Ibsi) …………… | [ ] 5 | |
| **C.** | **Beekumsaa fi mudannoo dhibuu Buujalee** | | | |
| **KT02** | Buujaleen maaliin nama qabachuu dandeessi? | Parasite/maxxantuu | [ ] 1 | |
| | | Witchcraft | [ ] 2 | |
| | | Cult | [ ] 3 | |
| | | Mental illness/ | [ ] 4 | |
| | | Worms/Raammoo | [ ] 5 | |
| | | Dirt/Dhukkee ykn kosii | [ ] 6 | |
| | | Hin beeku | [ ] 7 | |
| | | kan biroo(ibsi)……………. | [ ] 8 | |
| **KT03** | Maatiin keessan dhimma kana irratti maryattanii beektuu? | Eeyyeen | [ ] 1 | |
| | | Lakkii | [ ] 2 | |
| **KT04** | Buujaleen akka dhukkubatti ni ilaalamtii? | Eeyyeen | [ ] 1 | |
| | | Lakkii | [ ] 2 | |
| **KT05** | Buujaleen akkamtti ittisuun danda'ama? | ________________________________ | | |
| **KT06** | Ijoollee waggaa 5 hanga 14 jiran maatii sana keessaa filadhuu ilaali. Baatii sadan darban keessa bujaleen isaan mudachuu isaa qorachuun guuti. Mallattoolee ibsituu dhukkubichaa ilaali. Dhukkubichaan qabamanii turanii? | Eeyyeen | [ ] 1 | Yoo 1 ta'e gorsaa fi yaalumsa dandeessu godhiif. |
| | | Lakkii | [ ] 2 | |
| **KT07** | Umuriin mucaa ilaalte kun waggaa meeqa? | Waggaa_________ | | |
| **KT08** | Mucaan ilaalte kun dhiira moo dhalaadha? | Dhalaa | [ ] 1 | |
| | | Dhiira | [ ] 2 | |
| **KT09** | Mucaan ilaalte kun barumsa barachaa jiraa? | Lakki barachaa hin jiru | [ ] 1 | |
| | | Eeyyeen barachaa jira | [ ] 2 | |
| **KT10** | Mucaan ilaalte kophee ni fayyadamaa? (Ilaaluun guuti) | Eeyyeen kophee duudaa godhata | [ ] 1 | |
| | | Eeyyeen kophee banaa godhata | [ ] 2 | |
| | | Miilla qullaa deema | [ ] 3 | |
| | | Kan 6iro (ibsi)_______ | [ ] 4 | |
| **KT11** | Buujaleen yeroo isin mudatta maal gootu? | ________________________________ | | |
| **KT12** | Dhuukubni buujalee miidhaa caalu kan inni fidu/ kan rakkicha ammeessu maali ? | 'Teetanosii' | [ ] 1 | |
| | | Kulkula /madaa'uu | [ ] 2 | |
| | | hir'ina qaamaa/shafoo | [ ] 3 | |
| | | Kan 6iro (ibsi) …… | [ ] 4 | |
| **KT13** | Dhibee buujalee waktii irratti hundaa'aa? | Eeyyeen | [ ] 1 | |
| | | Lakkii | [ ] 2 | |
| **KT14** | Eeyyee yoo ta'e, yeroo rakkoon kun hammattu yoomi? | ________________________________<br>______________________________ | | |
| **KT15** | Nama buujalee qabu hawaasinni naannoo ni qooduu/ni balaaleffatuu? | Eeyyeen | [ ] 1 | |
| | | Lakkii | [ ] 2 | |

Galatoomaa, gaaffilee n keenya kanuma irratti xumurra.



Printed by BoltPDF (c) NCH Software. Free for non-commercial use only.
